# The Uncoupling Proteins: A Systematic Review on the Mechanism Used in the Prevention of Oxidative Stress

**DOI:** 10.3390/antiox11020322

**Published:** 2022-02-06

**Authors:** Jonathan Hirschenson, Emiliano Melgar-Bermudez, Ryan J. Mailloux

**Affiliations:** The School of Human Nutrition, Faculty of Agricultural and Environmental Sciences, McGill University, Sainte-Anne-de-Bellevue, QC H9X 3V9, Canada; jonathan.hirschenson@mcgill.ca (J.H.); emelgar@kerostx.com (E.M.-B.)

**Keywords:** uncoupling proteins, reactive oxygen species, mitochondria, proton leaks

## Abstract

Mitochondrial uncoupling proteins (UCP) 1-3 fulfill many physiological functions, ranging from non-shivering thermogenesis (UCP1) to glucose-stimulated insulin release (GSIS) and satiety signaling (UCP2) and muscle fuel metabolism (UCP3). Several studies have suggested that UCPs mediate these functions by facilitating proton return to the matrix. This would decrease protonic backpressure on the respiratory chain, lowering the production of hydrogen peroxide (H_2_O_2_), a second messenger. However, controlling mitochondrial H_2_O_2_ production to prevent oxidative stress by activating these leaks through these proteins is still enthusiastically debated. This is due to compelling evidence that UCP2/3 fulfill other function(s) and the inability to reproduce findings that UCP1-3 use inducible leaks to control reactive oxygen species (ROS) production. Further, other studies have found that UCP2/3 may serve as Ca^2+^. Therefore, we performed a systematic review aiming to summarize the results collected on the topic. A literature search using a list of curated keywords in Pubmed, BIOSIS Citation Index and Scopus was conducted. Potentially relevant references were screened, duplicate references eliminated, and then literature titles and abstracts were evaluated using Rayyan software. A total of 1101 eligible studies were identified for the review. From this total, 416 studies were evaluated based on our inclusion criteria. In general, most studies identified a role for UCPs in preventing oxidative stress, and in some cases, this may be related to the induction of leaks and lowering protonic backpressure on the respiratory chain. However, some studies also generated evidence that UCP2/3 may mitigate oxidative stress by transporting Ca^2+^ into the matrix, exporting lipid hydroperoxides, or by transporting C-4 metabolites. Additionally, some showed that activating UCP1 or 3 can increase mitochondrial ROS production, even though there is still augmented protection from oxidative stress. *Conclusion*: Overall, most available studies demonstrate that UCPs, particularly UCP2/3, prevent oxidative stress. However, the mechanism utilized to do so remains elusive and raises the question that UCP2/3 should be renamed, since they may still not be true “uncoupling proteins”.

## 1. Introduction

Oxidative phosphorylation (OXPHOS) relies on electron tunneling between several redox pairs in the respiratory complexes and the establishment of a protonmotive force (PMF), which powers complex V and the production of ATP. Electron flow through the chain is not perfectly coupled to OXPHOS, and electrons can prematurely “spin off” from the complexes and react with molecular oxygen (O_2_) to form reactive oxygen species (ROS) [1]. The proximal ROS formed by mitochondria is hydrogen peroxide (H_2_O_2_), which can cause oxidative stress at high enough levels, but it can also be used in cellular signaling, if its production is controlled [1]. The rate of mitochondrial ROS production is highly dependent on the polarity of the mitochondrial inner membrane (MIM). Additionally, there is a non-Ohmic relationship between PMF and ROS production [2]. Small increases in PMF can lead to an exponential increase in ROS production [2]. This is due to increased protonic backpressure on the chain and a slowing of mitochondrial respiration, which over-reduces redox pairs in the complexes, resulting in an increased availability of electrons for ROS production. This can lead to the induction of oxidative stress, culminating with cellular damage and cell death. Thus, although moderate mitochondrial ROS production can be beneficial, mechanisms inhibiting its excessive production can protect against oxidative stress.

UCP1 was first discovered over 40 years ago and was called “thermogenin” because of its role in non-shivering thermogenesis [3]. It was later renamed “the uncoupling protein” due to the finding that it bypasses complex V of the electron transport chain, returning protons to the matrix and releasing the Gibbs free energy stored in PMF as heat [3]. Several homologs of UCP1 were discovered later in 1996, and to date thousands of studies have been published on these proteins. UCP2 and 3 have ~73% homology with one another and both have ~58% homology to the thermogenic uncoupler, UCP1 [4]. UCP3 is almost exclusively expressed in skeletal muscle and, to a lesser extent, in brown fat [5]. UCP2, on the other hand, is ubiquitously expressed in several cell types and tissues [5]. Over-expression of UCP2 also plays a prominent role in cancer progression and resistance to chemotherapeutics by preventing oxidative stress and facilitating aerobic glycolysis and glutaminolysis [6,7]. 

UCP2 and 3 were originally thought to play a thermogenic role, much like UCP1. However, investigation into their functions revealed that both solute anion carriers prevent oxidative stress (reviewed in [8]). Use of GDP and fatty acids, which inhibit and activate the uncoupling proteins, respectively, revealed that UCP2 and 3 may prevent oxidative stress by decreasing mitochondrial ROS production through the return of protons to the matrix [9,10]. Furthermore, these studies suggested UCP1 may fulfill a similar role by using leaks during thermogenesis to mitigate ROS production in brown fat [10,11]. These findings generated much excitement, since it indicated that inducible proton leaks serve as an “emergency shut off valve” to inhibit mitochondrial ROS production [10,11]. To date, leaks through UCPs and other solute anion carriers, such as adenine nucleotide translocase (ANT), represent one of the most studied mechanisms for how mitochondria control ROS production. 

There is a consensus, to a certain degree, in the scientific community that UCPs, particularly UCP2/3, can protect against oxidative stress. However, the mechanisms by which this is achieved are still enthusiastically debated. Indeed, this controversy can be traced back to the finding that superoxide (O_2_^●−^) and the lipid hydroperoxide, 4-hydroxy-2-nonenal (4-HNE), are utilized as a negative feedback loop for their own production by directly activating proton leaks through UCP1-3 [10,11]. The controversies surrounding this mechanism have been addressed in several reviews, and there is compelling evidence that ROS and other electrophiles do not activate UCP1-3 [4,12,13]. To date, only UCP1 is accepted as the true “uncoupler”, leaving some to suggest that UCP2/3 were erroneously named “uncoupling proteins” (reviewed in [4]). Furthermore, several groups have found evidence that UCP2 and 3 do not leak protons and instead prevent oxidative stress by transporting calcium (Ca^2+^) and C4 metabolites, such as oxaloacetate or hydroperoxides [7,14,15]. The debate and troubled history surrounding UCP2/3 in preventing oxidative stress can be best highlighted by a recent publication detailing the complications associated with measuring leaks through these proteins and the underlying bioenergetics justifying why they do not participate in proton return [4]. Furthermore, this comprehensive review also details why UCP2/3 may not be able to fulfill important physiological functions, such as glucose-stimulated insulin secretion [4]. Based on the enthusiastic debate surrounding the use of proton leaks through UCP1-3 to reduce mitochondrial ROS, we decided to perform the first-ever systematic review of the known literature invested in elucidating the mechanism by which these proteins prevent oxidative stress.

## 2. Methods

### 2.1. Search Strategy and Eligibility Criteria

The systematic search was designed in accordance with Preferred Reporting Items for Systematic Reviews and Meta-analyses (PRISMA) guidelines [16]. The protocol was registered with the Open Science Framework (OFR) (https://osf.io/zqxd6, accessed on 19 January 2022). The search strategy was initiated by formulating the research question “Does UCP1-3 prevent oxidative stress by inhibiting ROS production?”. This was utilized to formulate a list of keywords and keyword combinations (Table 1) for literature searches. Keywords and keyword combinations were utilized to extract records from PubMed, SCOPUS and BIOSIS Citation Index, respectively.

Inclusion criteria: Experimental approaches utilized to assess the biochemical function(s) of UCP1-3. Inclusion criteria included studies carried out using UCPs inserted into liposomes, human or rodent UCPs expressed in *S. cerevisiae*, studies utilizing isolated mitochondria, cell lines and primary rodent or human cells, or permeabilized muscle fibers and other tissue types. Rodent and mammalian cell line or primary cell models where UCPs were either knocked out or over-expressed were also included. Studies that directly measured the functionality of UCP1-3, including polarographic or fluorometric measurements of respiration, role of UCPs in limiting or increasing ROS production and preventing/promoting oxidative stress, patch-clamp studies, membrane potential measurements, solute transport and structural studies, articles that measured UCP mRNA or protein expression in response to electrophilic and environmental stress and reports that investigated the physiological functions of UCPs in the contexts of the biochemical functions listed above, were also part of the inclusion criteria. Human studies where variants of the UCP gene were examined in the context of the criteria listed above were also included. Studies that measured UCP1-3 expression without conducting any biochemical and mechanistic studies listed above or assessed the function of UCP homologs in non-mammals were excluded from the review. Original research communications, reviews and short communications were considered.

Exclusion criteria: Studies conducted on UCP4 and 5, or UCP homologs in non-mammals (the literature search would have been too broad), and articles published in predatory journals (therefore, we consulted Beall’s list) were excluded, and only articles published in English were considered. Hypothesis articles were also not considered. Only studies published from 1997 onward were considered, and magazine articles and conference proceedings were excluded.

Titles and abstracts were screened by J.H. and R.J.M. using PubMed, SCOPUS and BIOSIS for the keywords listed above. In some cases, hand searches of the articles or reference lists were conducted to identify keywords and keyword combinations. Duplicate reports were eliminated, and the included records were imported into Rayyan QCRI Intelligent Systematic review. Inclusion and exclusion criteria were utilized by J.H., E.M.B. and R.J.M. to determine which studies should be utilized for the review. Conflicts in the identified studies were resolved by all three authors in several Zoom sessions. J.H., E.M.B. and R.J.M resolved conflicts using the inclusion and exclusion criteria and hand searches of the record.

### 2.2. Data Extraction: Data from the Included Studies Were Extracted from Rayyan and Itemized in Excel

Data were extracted by J.H. and E.M.B. The extracted data were then re-evaluated by J.H. and E.M.B. R.J.M., who resolved disagreements by referencing the original publication. Information extracted from each study was as follows: (1) characteristics of the publication (publication date, journal the article was published in and study design, (2) mechanisms for UCP1-3 function were interrogated using accepted techniques for the study of bioenergetics, (3) UCP1-3 gene expression and its relationship with cell responses and physiological functions and (4) study models: use of purified proteins inserted into vesicles, isolated mitochondria, cell culture (primary rodent and human or cell lines over-expressing/under-expressing UCP1-3) and whole animal models or human volunteer studies. Studies that over-expressed mammalian UCPs in yeast models or non-mammals were also considered.

## 3. Results and Discussion

### 3.1. Literature Search and Characteristics of Eligible Studies

**Figure 1:**
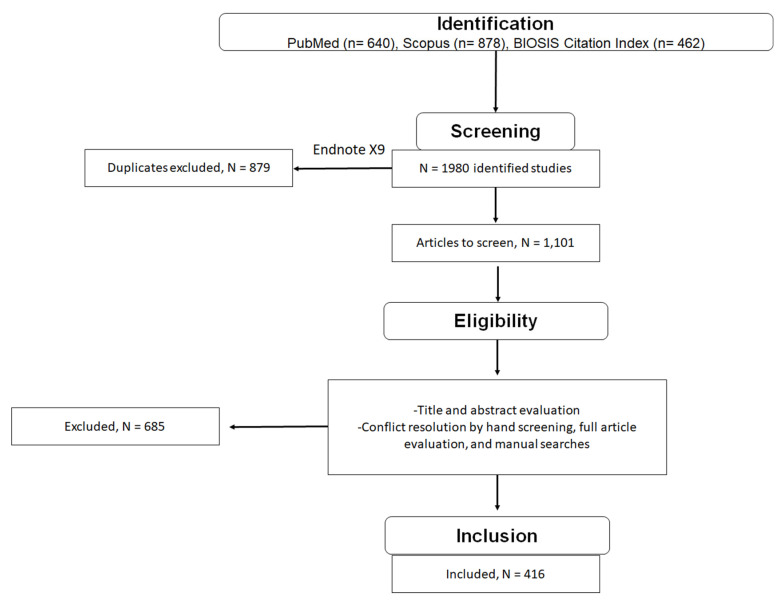
Flow chart depicting the literature screening method.

**Figure 2:**
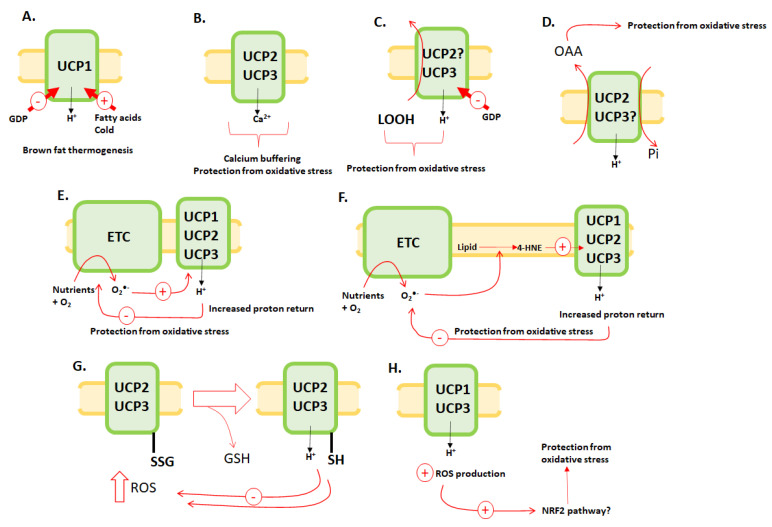
The suggested mechanisms for UCP1-3-mediated prevention of oxidative stress. It is important to note the only accepted mechanism for proton return occurs through UCP1 in brown fat for thermogenesis. (**A**) The accepted mechanism for the activation of proton leaks through UCP1 for brown fat thermogenesis. Cold exposure and fatty acids activate proton return through UCP1, whereas the nucleotide, GDP, has the opposite effect. Cold-mediated activation requires norepinephrine release by the sympathetic nervous system surrounding brown adipose tissue. (**B**) UCP2/3 may serve as a mitochondrial calcium uniporter or activate mitochondria Ca^2+^ uptake 1 (MICU-1) following its methylation. (**C**) UCP3 and potentially UCP2 may mitigate oxidative stress by exporting lipid hydroperoxides into the cytoplasm. (**D**) UCP2 and potentially UCP3 may serve as an antiporter for the maintenance of aerobic glycolysis and glutaminolysis. The added benefit is the export of C4 metabolites oxaloacetate (OAA) or aspartate (not in diagram for clarity) into the cytosol, which are converted to malate for NADPH production and prevention of oxidative stress. (**E**) Superoxide (O_2_^●−^) mitigates its own production by the electron transport chain (ETC) by activating proton return through UCP1-3, lowering protonic backpressure on the chain and preventing oxidative stress. (**F**) O_2_^●−^ production by an over-reduced ETC induces lipid peroxidation and the genesis of 4-hydroxy-2-nonenal (4-HNE). 4-HNE activates leaks through UCP1-3, lowering protonic backpressure on the chain, mitigating oxidative stress. (**G**) UCP2/3 are deactivated by glutathionylation. Oxidation of GSH pools due to a spike in ROS deglutathionylates UCP2/3 activating leaks to prevent oxidative stress. (**H**) Activating leaks through UCP1/3 increases mitochondrial ROS production due to the augmentation of fuel burning for either thermogenesis (UCP1) or the metabolism of excess fats, following exposure to a high-fat diet (UCP3). The increase in ROS production has been linked to increased resistance to oxidative stress and higher antioxidant defenses. The increase in ROS may activate the NRF2 signaling pathway to elicit this effect.

The strategy used for the keyword search, screening and record inclusion is shown in Figure 1. In total, 640, 878 and 462 studies were identified with PubMed, SCOPUS and BIOSIS, respectively, using the keywords in Table 1. From this, 1980 potential references were screened by title and abstract scanning and hand searches. A total of 879 duplicate searches were excluded, and the remaining 1101 studies were imported into Rayyan. Assessment of the records resulted in the exclusion of 685 articles from the review. Therefore, a total of 416 records fulfilled the eligibility criteria. Notably, we did not cite all 416 studies and reviews below. However, the enl file that contains all 416 records has been included. To date, the only accepted mechanism for proton translocation occurs through UCP1 in response to cold exposure and thus plays a vital role in thermogenesis (Figure 2A). By contrast, the mechanisms by which UCP2/3 prevent oxidative stress is summarized in Figure 2. This summary is based on the literature collected from our keyword searches. Note that none of these mechanisms have been formally accepted by the field and remain to be verified. UCP1 has also been suggested to participate in some of these pathways for prevention of oxidative stress (Figure 1). 

### 3.2. UCP2 and 3 and the Transport of Calcium

Mitochondrial Ca^2+^ is vital for the regulation of OXPHOS, cell signaling and the induction of apoptosis. The disruption of cell Ca^2+^ homeostasis is associated with the induction of oxidative stress, and mitochondria are integral for maintaining this homeostasis. Yet, the identity of the mitochondrial Ca^2+^ transporter remained elusive for many years. In 2008, Trenker et al. discovered that mitochondrial Ca^2+^ uptake occurs by a uniport mechanism driven by UCP2 and 3 [14]. A total of 21 articles were found that investigated the relationships between UCP2/3 function and Ca^2+^ homeostasis (please see the appended enl file). Only a handful of these investigations studied UCP2/3-mediated transport of Ca^2+^. Trenker et al. demonstrated that mitochondrial Ca^2+^ uptake declined significantly when UCP2 and UCP3 were knocked down in cultured cells and in mitochondria from mice homozygous for UCP*2* gene (Figure 2B) [14]. These findings were confirmed by using cell systems that over-expressed UCP2 and 3 [14]. Furthermore, their findings suggested that no pre-activation of UCP orthologs was required to trigger Ca^2+^ sequestration [14]. Mutation of an A-R-E-E domain that sites in the second inter-membrane spanning region, which forms a helix that contacts the matrix, in UCP2 and 3 inhibited mitochondrial Ca^2+^ uptake [14]. Overall, the authors concluded that UCP2/3 are the long-sought-after and elusive mitochondrial Ca^2+^ uniporter (MCU). 

Reports of UCP2/3 contributing to mitochondrial Ca^2+^ uptake generated much excitement in the field, and attempts to replicate these findings were conducted. Brookes et al. used isolated rat liver and heart mitochondria, which reportedly express UCP2 and UCP3, respectively, but were unsuccessful in replicating these findings [17]. The incubation of these mitochondria in genipin and GDP, inhibitors for both uncoupling proteins, did not interfere with mitochondrial Ca^2+^ uptake [17]. Furthermore, conducting similar experiments with skeletal muscle mitochondria isolated from mice, containing a deletion for UCP*2* or *Ucp3* gene, did not result in any alteration in Ca^2+^ uptake [17]. Coupled with the established knowledge on the impact of mitochondrial bioenergetics and PMF on Ca^2+^ uptake, it was concluded by the authors that the interpretation of the results collected by Trenker et al. should be met with caution. This scrutiny was met with a rebuttal published by Trenker et al. that re-examined the concerns raised in [18]. In their response, Trenker et al. were successful in demonstrating that over-expression of UCP2/3 does augment the kinetics for Ca^2+^ uptake and that silencing the genes encoding both proteins had the opposite effect [18]. 

Several studies from the same group showed UCP2/3 are required for Ca^2+^ uniport (reviewed in [19]). However, others provided no evidence that they fulfill this function, raising questions as to whether these uncoupling proteins are Ca^2+^ transporters [17,20]. A breakthrough in identifying the elusive mitochondrial Ca^2+^ transporter came with the discovery of the protein mitochondria Ca^2+^ uptake 1 (MICU-1), an essential component of the Ca^2+^ transporter [21]. Other key components of the mitochondrial Ca^2+^ uniporter, which will not be discussed here, were also identified later [21]. It was later found that protein arginine methyl transferase 1 (PRMT1)-mediated methylation of MICU-1 reduces Ca^2+^ uptake, which can be reversed by UCP2 binding to the methylated MICU-1 [21]. Taken together, whether UCP2/3 facilitate Ca^2+^ uptake to prevent oxidative stress is inconclusive. Overall, the function of UCP2/3 in the transport of Ca^2+^ has not been accepted by the field. 

### 3.3. UCP2 and 3 in the Transport of Lipid Hydroperoxides

A total of 16 articles that investigated relationships between UCP2/3 expression and lipid hydroperoxides metabolism were identified using our keyword search for “lipid hydroperoxides” and “4-hydroxy-2-noneal or 4-HNE” in the included studies. All 16 of these articles examined the role of lipid hydroperoxides and 4-HNE in the activation of proton return through UCP1-3 (see enl file). It was reported in most of these studies that lipid hydroperoxides and 4-HNE activate leaks through UCP1-3 to inhibit ROS production and protect from oxidative stress (see enl file). It is notable that some studies used UCP2 over-expressed in liposomes derived from *E. coli* [22], which was later concluded to induce an artificial increase in proton return due to increased leakiness of membranes. This was also demonstrated in cases where studies over-expressed UCP3 by up to ~20-fold [4]. Other studies were unable to repeat findings collected showing 4-HNE activates leaks, which is discussed further below. Thus, the mechanism for UCP1-3 mediated prevention of oxidative stress remained elusive (and some argue that it is still undefined [4]). To identify how UCPs prevent oxidative stress, one study showed that UCP3 exports lipid hydroperoxides from the matrix of mitochondria to protect it from oxidative stress. Lombardi et al. utilized skeletal muscle mitochondria isolated from wild-type and *Ucp3*-null mice to show UCP3 is a lipid hydroperoxide exporter [15]. The study focused on utilizing arachidonic acid (AA) to stimulate proton conductance through UCP3 [15]. Using this model system, Lombardi et al. provided evidence that activated UCP3 exports lipid hydroperoxides (Figure 2C) [15]. This transport was dependent on the production of O_2_^●−^ and the subsequent genesis of lipid hydroperoxides on the inner leaflet of the MIM [15]. Although the evidence presented in this article is compelling, it has not been reproduced by other groups and, therefore, has not been accepted by the field. 

### 3.4. UCP2 Transports C4 Metabolites to Prevent Oxidative Stress

Hypothesis articles were omitted from the methodology used in this systematic review. However, discussing a few in this section is relevant, since it was proposed that UCP2/3 could translocate substrates for energy metabolism [23], which was later found to be the case for UCP2 [7]. The quest to identify how UCP2/3 prevent oxidative stress led to the genesis of several hypotheses that both solute anion carrier family members transport metabolites to prevent oxidative stress and maintain the efficiency of energy metabolism. For example, it was proposed that UCP3 serves as a fatty acid “cycler” that removes fatty acids from the matrix to facilitate the more efficient beta oxidation of acyl-CoA [24]. This hypothesis was based on observations that activation of UCP3 promotes weight loss or induces an “exercise-like” effect by promoting fatty acid oxidation in muscles of rodents and humans [25,26]. It was later disproven by a study demonstrating that UCP3 does not transport fatty acids [27]. 

During the same period, it was also proposed that UCP2/3 and the avian homolog may play a part in pyruvate and glutamine metabolism [23]. This was based on the observation that (1) UCP3 is more highly expressed in glycolytic muscle fibers and serves as a passive pyruvate translocator to maintain equilibrium between glycolysis and oxidative phosphorylation and (2) that glutamine induces the expression of UCP2 [23]. Vozza et al. later found that UCP2 serves as a dicarboxylate carrier and exchanges mitochondrial C4 metabolites, specifically oxaloacetate or aspartate, for cytosolic inorganic phosphate using an H^+^-assisted system that requires both the electrical and pH-dependent components of the protonmotive force (Figure 2D) [7]. The export of either C4 metabolite was found to be integral for driving glutaminolysis while simultaneously promoting the conversion of pyruvate generated by glycolysis to lactate, which was proposed to partake in the Warburg effect and drive cancer cell metabolism [7]. Intriguingly, in the same study, the authors found that knocking down UCP*2* gene in cell culture also led to the oxidation of glutathione pools [7]. This implies this transport mechanism also plays a part in maintaining the redox buffering capacity of the glutathione pool, which would protect cells from oxidative stress. Based on the proposed mechanism, this can be achieved by the conversion of aspartate to oxaloacetate and then malate in the cytosol [7]. This is followed by the oxidative decarboxylation of malate to pyruvate by malic enzyme and the production of NADPH for the reduction of GSSG to GSH [7]. Overall, these findings present a novel proposed function for UCP2 and imply that it may prevent oxidative stress by exporting C4 metabolites from the matrix of mitochondria. 

### 3.5. UCP1-3 Preventing Oxidative Stress by Limiting ROS Production: A Controversial Mechanism

We identified 385 studies related to UCP1-3 preventing oxidative stress by limiting ROS production (see enl file). Some of these studies interrogated this function in the context of activating proton return through UCP1-3 to decrease protonic backpressure on the respiratory complexes, which lowers the number of electrons available for the premature reaction with O_2_ to make ROS. The discovery of UCP1 isoforms, UCP2 and UCP3, led to the development of the hypothesis that solute anion carrier family members also play a part in thermogenesis. However, it was later found that this was not the case, even though in a handful of studies it was shown that treating rodents with 3,4-Methylenedioxymethamphetamine induced UCP3-dependent production of heat [28]. The physiological function of UCP2/3, therefore, remained elusive. However, in 1997, it had been established that there is a non-Ohmic relationship between membrane potential and the rate of mitochondrial ROS production [2]. In the same year, Negre-Salvayre et al. interrogated the potential function of UCP2 in the prevention of mitochondrial ROS production and found that GDP, an uncoupling protein inhibitor, augmented H_2_O_2_ generation by preventing proton return to the matrix [9]. Together with previously published literature showing induction of leaks with protonophores can lower ROS production, it was proposed that the function of UCPs was to decrease mitochondrial ROS production by “leaking” protons back to the matrix, reducing protonic backpressure on the electron transport chain [29]. 

These exciting findings were then applied to studying how UCP1-3 can protect cells from oxidative stress by lowering mitochondrial ROS production. Mitochondria use ROS, specifically H_2_O_2_, for cell signaling as well. Therefore, cells need to strike a delicate balance between promoting bursts in mitochondrial ROS production while protecting cells from its potential toxicity. This meant that there needed to be a “safety valve” in place to curtail production once there is a burst in ROS in response to cell stimuli. In 2002, a study interrogating inducible leaks through the uncoupling proteins found O_2_^●−^ activates UCP1-3 (Figure 2E) [10]. Further, induction by O_2_^●−^ requires fatty acids and is inhibited by GDP [10]. The same group later found this was achieved through the O_2_^●−^ mediated production of carbon-centered lipid radicals and the formation of 4-HNE (Figure 2F) [11]. Unfortunately, other groups failed to reproduce these findings, and Echtay et al. never provided evidence that 4-HNE is generated, leaving this mechanism for how UCPs prevent oxidative stress unaccepted. Deglutathionylation of UCP2/3 has also been suggested to be the mechanism for the activation of leaks through these proteins for the prevention of mitochondrial ROS production (Figure 2G). This was based on several studies using purified proteins, cells and mitochondria isolated from wild-type, *Ucp3−/−*, and *Grx2+/−* and *Grx2−/−* mice, cell lines over-expressing wild-type or UCP3 variants containing mutations for Cys and cells knocked down for GRX2 [30,31]. However, this other suggested mechanism has not been accepted by the field either.

Multiple investigations have found activated UCP2/3 prevents oxidative stress. In some studies, this has been related to an increase in state 4 respiration, a proxy measure for proton leaks, and a decrease in ROS production. The capacity for UCP2/3 to fulfill this function has been related to some physiological functions and has led to the suggestion these proteins may be targets for the treatment of metabolic disorders. For example, it was found the induction of leaks through UCP2 plays an integral role in insulin release [32]. Similarly, increased leaks through UCP3 have been linked to resistance to diet-induced obesity in rodent models and more efficient weight loss in human volunteers [25,26]. These findings led to the suggestion that UCP3 could be a pharmacological target for the treatment of metabolic disorders [29]. Although many studies have been able to measure UCP2/3-dependent increases in state 4 respiration, either after their activation with fatty acids, interactions with O_2_^●−^ or 4-HNE, or following deglutathionylation, some other studies have been unable to demonstrate UCP2/3 can increase non-phosphorylating respiration [33,34]. The proton leak mechanism for UCP1 has been accepted by the field, and, thus, UCP2/3 were suggested to utilize a similar proton conductance mechanism to fulfill their physiological functions(s). The caveat to this suggested mechanism is that UCP2/3 are expressed at several orders of magnitude lower in some tissues (e.g., 4 pmol UCP3/mg skeletal muscle mitochondrial protein or 0.1–0.4 pmol UCP2/mg of INS-1E or kidney mitochondrial protein) when compared to UCP1 (100–1000 pmol/mg mitochondrial protein; note this range depends on whether brown fat is active or not) [4]. This implies that UCP2/3 must have a much higher rate of proton conductance when compared to UCP1 [4]. Overall, there is a lot of evidence in favor of the use of a proton leak mechanism by UCPs to lower ROS production. However, this mechanism is also not accepted by the field due to several studies that failed to demonstrate UCP2/3 leak protons decrease ROS production and questions surrounding their expression level in comparison to UCP1. Further, it is also not accepted that UCP1 curtails ROS production. 

### 3.6. Studies Showing UCP1/3 May Augment Mitochondrial ROS Production

As discussed above, many studies have shown a direct relationship between UCP2/3 activity and expression and the prevention of oxidative stress, which is achieved by lowering mitochondrial ROS production. This mechanism was also suggested for UCP1 by a few studies. Intriguingly, four studies showed that activating proton leaks can increase ROS production (Figure 2H). Mailloux et al. showed in 2012 that adding oleate, a UCP1 activator, to actively respiring brown adipose mitochondria resulted in a significant increase in O_2_^●−^/H_2_O_2_ production, which was mirrored by the augmentation of proton leaks [35]. Additionally, it was found that cold exposure augmented ROS production by these mitochondria, which was increased further after exposure to oleate [35]. These findings correlated with the depolarization of mitochondria and a corresponding increase in respiration [35]. Shabalina et al. made a similar observation in 2014 [36]. Indeed, cold exposure resulted in an increase in ROS production by brown fat mitochondria energized with palmitoyl-CoA or glycerol-3-phosphate and treated with or without rotenone [36]. The authors also utilized a UCP1 knockout line to show there is no correlation between its expression and activation and the production of ROS [36]. Collectively, Shabalina et al. concluded that UCP1 activation increases mitochondrial ROS production and extrapolated their findings to speculate that depolarization of the membrane potential may not lead to a corresponding decline in O_2_^●−^/H_2_O_2_ generation [36]. A 2016 study published by Chouchani et al. successfully showed that cold acclimation in rodents was associated with a corresponding increase in UCP1 activation and mitochondrial ROS production [37]. Intriguingly, the authors also observed that the H_2_O_2_-mediated oxidation of Cys253 in UCP1 to a sulfenic acid is required for the induction of thermogenesis [37]. This would imply that the sharp rise in H_2_O_2_ production plays a role in brown fat thermogenesis through the sulfenylation of UCP1 [37]. The only caveat to these findings is that H_2_O_2_ reacts slowly with most cellular protein cysteine thiols and that sulfenic acid moieties are highly unstable and can form irreversible adducts with lipid hydroperoxides or undergo over-oxidation. Finally, a recent study demonstrated that promoting deglutationylation in mouse muscles protects from diet-induced obesity by augmenting fuel combustion that is associated with the activation of leaks through UCP3 and ANT [25]. This was associated with an increase in mitochondrial ROS production and a corresponding increase in resistance to oxidative stress [25]. It was suggested that the increased redox buffering capacity in these muscles may be related to the activation of the NRF2 signaling pathway, which would bolster antioxidant defenses and mechanisms for the prevention of oxidative stress. However, it should be noted again that the capacity of UCP1/3 to activate ROS production for adaptative signaling has not been interrogated and is, at this point, speculative at best.

The four studies described above that interrogated the relationship between UCP1 and ROS production provide evidence that its activation can augment H_2_O_2_ by mitochondria. This led to questions as to whether proton leaks and decreasing protonic backpressure can serve to limit ROS production. However, there are several important distinctions to address regarding the impact of leaks on the bioenergetics of mitochondria from brown fat in comparison to other tissues. One notable difference is that UCP1 makes up 100–1000 pmol/mg mitochondrial protein brown adipose tissue [4]. This contrasts with other tissues where UCP2 and 3 expression account for 0.1–5 pmol/mg of total mitochondrial protein [4]. Therefore, the activation of UCP1 in brown fat leads to a rapid and sharp depolarization of the membrane potential, which is offset by a several-magnitude increase in respiration and energy expenditure. The rapid burning of fuels following UCP1 activation could account for the increase in ROS production. What is counter intuitive, however, is the relationship between UCP3 “activation” in muscles and the increase in ROS production due to a four-fold increase in fatty acid oxidation. It should be noted first that this was reported to be due to the loss of one of the two genes encoding *Grx2*, leading to UCP3 deglutationylation and activation [25]. There are still some discrepancies with this suggested mechanism, including the low expression level of UCP3 in muscle when compared to UCP1 in brown fat. It could be explained by the simultaneous activation of leaks through ANT, which is more abundant than UCP3. This raises questions, though, about the importance of UCP3 in contributing to proton leaks in mitochondria. Overall, only four studies showed UCP1 and 3 activation increase ROS production, which may bolster antioxidant defenses through NRF2 signaling. Thus, this mechanism for regulating ROS availability and preventing oxidative stress is far from being accepted by the scientific community.

## 4. Conclusions

After 40 years, the induction of non-shivering thermogenesis in brown fat through the activation of proton leaks through UCP1 is widely accepted by the field. Decreasing the mitochondrial membrane potential to diminish protonic backpressure on the electron transport chain is also an undisputed function of proton leaks, as simulated with protonophores. The discovery of the so-called “novel uncoupling proteins”, UCP2 and UCP3 (designated as SLC25A8 and SLC25A9), eventually led to the genesis of the postulate that mammalian and non-mammalian cells express inducible “uncouplers” that utilize proton return to the matrix to decrease mitochondrial ROS production. Many studies focused on this concept and successfully showed that the uncoupling proteins, mainly UCP2/3, are required to prevent oxidative stress. However, their capacity to do so by decreasing ROS production using proton leaks is still a subject of enthusiastic debate over 20 years later. 

Here, using keyword searches and a systematic review protocol, we attempted to shed new light on the mechanism used by UCP1-3 to prevent oxidative stress. We identified 416 articles and reviews that interrogated these mechanisms. Several articles supplied compelling evidence that UCP2/3 may prevent oxidative stress by transporting Ca^2+^, lipid hydroperoxides or C4 metabolites. Many studies provided strong evidence that protection from oxidative stress and limiting ROS production is achieved through a proton leak mechanism. However, as noted above, these observations must be interpreted with caution. Indeed, four studies found UCP1 and 3 augment ROS production, potentially due to a several-fold increase in fuel metabolism in mitochondria. Additionally, careful considerations must be made regarding the methods and model systems used when interpreting findings regarding the function of UCP1-3 in the prevention of oxidative stress by using a proton leak mechanism, which was elegantly reviewed in [4]. Based on this systematic review, we conclude that UCP2/3, and potentially UCP1, prevent oxidative stress by limiting ROS production. However, we also conclude that the mechanism by which UCP2/3 do so remains elusive. Finally, we cannot exclude the possibility that UCP2/3 fulfills cell function(s) outside of controlling the availability of ROS, such as through Ca^2+^ or metabolite transport. 

## Figures and Tables

**Table 1:** Keyword search and word combinations used to determine whether uncoupling proteins (UCP) use leaks to control oxygen species (ROS).

Uncoupling Proteins	Reactive Oxygen Species	Fuel Metabolism
UCP1 UCP2 UCP3 uncoupling protein 1 uncoupling protein 2 uncoupling protein 3	ROS Glutathionylation lipid hydroperoxide superoxide 4-hydroxy-2-nonenal hydroxynonenal	fatty acid oxidation fatty acid export fatty acid transport

(UCP1 OR UCP2 OR UCP3 OR “uncoupling protein 1” OR “uncoupling protein 2” OR “uncoupling protein 3” OR “uncoupling protein one” OR “uncoupling protein 2” OR “uncoupling protein 3”) AND (“reactive oxygen species” OR ROS OR glutathionylation OR “lipid hydroperoxide” OR superoxide OR 4-hydroxy-2-nonenal OR hydroxynonenal) AND (“oxidative stress” OR “fatty acid oxidation” OR “fatty acid export” OR “fatty acid transport”).

## Data Availability

The data is contained within the article.
